# Impact of Prolonged Fasting and Refeeding on Enteroendocrine Hormone Expression: One More Piece of the Fasting/Refeeding Metabolic Puzzle

**DOI:** 10.3390/biomedicines13092088

**Published:** 2025-08-27

**Authors:** Gonçalo Nunes, Marta Guimarães, Sofia B. Oliveira, Sofia S. Pereira, Francisco Vara-Luiz, Ivo Mendes, Carolina Palma, Cátia Oliveira, Jorge Fonseca

**Affiliations:** 1 Gastroenterology Department, GENE—Artificial Feeding Team, Hospital Garcia de Orta, 2805-267 Almada, Portugal; 2 ICBAS-UP—School of Medicine and Biomedical Sciences Abel Salazar, University of Porto, 4050-313 Porto, Portugal; 3 Aging Lab, Egas Moniz Center for Interdisciplinary Research (CiiEM), Egas Moniz School of Health and Science, 2829-511 Almada, Portugal; 4 UMIB—Unit for Multidisciplinary Research in Biomedicine, ICBAS—School of Medicine and Biomedical Sciences, University of Porto, Rua Jorge Viterbo Ferreira 228, 4050-313 Porto, Portugal; 5 ITR—Laboratory of Integrative and Translocation Research in Population Health, Rua das Taipas 135, 4050-600 Porto, Portugal

**Keywords:** gastrointestinal hormones, incretins, refeeding syndrome, enteral feeding, gastrostomy

## Abstract

**Introduction:** Prolonged fasting induces histological and ultrastructural changes of the intestinal mucosa that may reduce absorption in malnourished patients with high risk of refeeding syndrome. Endocrine function of the intestinal mucosa may be affected by starvation with potential implications for nutritional support. **Objective:** The present study aims to evaluate the expression of gastrointestinal hormones in duodenal enteroendocrine cells (EECs) of patients after a long starvation period and to assess the changes in EEC hormonal expression after enteral refeeding in the same individuals. **Methods:** This was an observational prospective controlled study. Adult patients submitted to endoscopic gastrostomy (PEG) with an ingestion below 50% of daily needs for at least one month were enrolled. Duodenal biopsies were collected before gastrostomy (T0) and after 3–6 months of PEG feeding (T1). Biopsies underwent immunohistochemical analysis for chromogranin-A (CgA), neurotensin and incretin (GLP-1 and GIP) tissue expression. Normal duodenum biopsies were used as controls. **Results:** A total of 30 patients (16 men/14 women) aged 67.1 ± 13.5 years were included, and 14 patients completed follow-up at both periods (46.7%). Malnutrition was diagnosed in all patients according to GLIM criteria. T0 tissue expression defined by median stained area for CgA, GLP-1, and GIP were significantly higher in patients compared to controls (CgA: 1.04% vs. 0.41%; GLP-1: 0.17% vs. 0.03%; GIP: 0.19% vs. 0.03%) (*p* < 0.001) without differences for neurotensin (0.01%) (*p* = 0.96). T1 hormonal tissue expression was not significantly reduced after 3–6 months of enteral refeeding (*p* > 0.05). **Conclusions:** Prolonged fasting induces increased expression of incretins and chromogranin-A in the duodenum that probably reflect an adaptative response to maintain the anabolic insulin effect under nutritional deficiency. Hormonal expression does not normalize after PEG refeeding during a short period.

## 1. Introduction

Protein-energy malnutrition is a highly prevalent condition associated with adverse functional and clinical outcomes. Acute and chronic inflammation plays a central role in disease-related malnutrition, leading to decreased food intake and muscle catabolism. Recent advances in clinical and artificial nutrition observed during the last decades have allowed an effective and safe implementation of nutritional therapy in different inflammatory and fasting clinical settings [1]. Nutritional assessment is highly recommended by international societies to avoid the negative consequences of prolonged fasting and the potential risks for nutritional support [2].

Refeeding syndrome (RS) is a potential life-threatening condition caused by metabolic and electrolyte disturbances, occurring alongside the reintroduction of nutrition in predisposed patients exposed to long-term caloric deficiency [3,4]. The pathophysiology of RS involves a dysregulated response to the sudden reactivation of anabolism caused by insulin secretion in patients with chronic adaptative low metabolic rate induced by prolonged fasting. Enteral feeding is commonly the first-line approach for managing malnutrition when the gastrointestinal system remains viable [2,3].

Patients undergoing percutaneous endoscopic gastrostomy (PEG) for long-term enteral feeding due to chronic disease with prolonged dysphagia are at particularly high risk; nevertheless, clinical and laboratory signs of RS are seldom identified in PEG-fed patients [5]. A recent prospective controlled study analyzed duodenal samples of patients referred for PEG and confirmed that starvation induces histological and ultrastructural changes in duodenal mucosa, which include shortening of the villi with or without mucosal atrophy and ultrastructural changes in individual enterocytes. These changes could reflect impaired absorption, which may be responsible for a relatively low prevalence of RS observed in the early post-PEG period. The study also demonstrated that after a period of enteral refeeding, enteral nutrition not only improves nutritional status but also normalizes the duodenal histology and ultrastructure [6].

Gastrointestinal hormones are a heterogenous group of polypeptides secreted by enteroendocrine cells (EECs) widely distributed throughout the mucosa of the digestive tract. These hormones regulate intestinal and pancreatic functions and contribute to digestive homeostasis by affecting secretion, motility, absorption, digestion, and cell proliferation [7].

Chromogranin-A (CgA) is one of the most abundant neuroendocrine secretory proteins localized in the secretory granules of the EEC in the gastrointestinal tract, being also secreted in other tissues such as the pancreas, adrenal medulla, and pulmonary alveoli. CgA is a precursor of several functional peptides that regulate a wide range of physiological processes including pancreatic secretion, cardiovascular function, and immune response [8].

Neurotensin is also produced by EEC despite being mainly distributed throughout the central nervous system where it is involved in analgesia, sleep control, thermoregulation and pituitary hormone secretion. Its digestive functions include stimulation of pancreatic and biliary secretions, inhibition of gastric acid secretion and motility, stimulation of colon motility and inhibition of jejuno-ileum motility favoring absorption. It also promotes the growth of normal gastrointestinal tissues [9].

Glucagon-like peptide 1 (GLP-1) and Gastric inhibitory polypeptide (GIP) are the two primary incretin hormones secreted from the intestine after ingestion of glucose or other nutrients to stimulate insulin secretion from pancreatic beta cells. In addition to their insulinotropic effects, GIP and GLP-1 play critical roles in several biological processes in different tissues. Within the pancreas, GIP and GLP-1 together also promote beta cell proliferation and inhibit apoptosis, but while GIP enhances postprandial glucagon response, GLP-1 suppresses it. In adipose tissues, GIP but not GLP-1 facilitates fat deposition. In bone, GIP induces bone formation while GLP-1 inhibits bone reabsorption. In the brain, both GIP and GLP-1 are thought to be involved in memory formation and appetite control [10,11].

The chronic decrease of nutrient load into the intestinal lumen has a high potential to impact the mucosa hormonal secretion. The present study of tissue expression of gastrointestinal hormones will contribute to understanding the metabolic shifts between long-term fasting and adequate feeding, as well as the complex functional mechanisms behind the previously described histological and ultrastructural changes induced by prolonged fasting and the recovery observed after enteral refeeding.

The authors aim to analyze the intestinal tissue expression of gastrointestinal hormones in malnourished patients when submitted to endoscopic gastrostomy after a significant period of fasting and to assess the hormonal expression pattern after enteral refeeding.

## 2. Material and Methods

### 2.1. Study Design

This single center, observational, and prospective study was conducted in a tertiary hospital. The present study was approved by the institutional ethics committee.

### 2.2. Patients

Consecutive ambulatory adult patients (≥18 years) with oral ingestion below 50% of energy daily needs for a minimum period of 1 month that were referred to PEG for long-term enteral nutrition were initially eligible. For each patient enrolled, the following methodology was applied: 

1. Nutritional anamnesis performed by a trained dietitian through a dietary recall.

2. Estimation of the energy needs according to age, gender, height, weight, and clinical setting to assess the reduction of food ingestion in the last month before PEG.

3. Prospective clinical and demographic database registry that includes age, gender, clinical indication for PEG, anthropometry, laboratorial parameters, and survival after the gastrostomy.

4. PEG performed by two gastroenterologists using the “pull” method described by Gauderer–Ponsky with patients under deep sedation. Antithrombotic therapy was managed according to international guidelines [12].

5. Collection of 4–6 duodenal biopsies (second portion) at the time of the gastrostomy procedure (T0) and after 3–6 months of PEG-feeding at the moment of endoscopic tube replacement (T1).

6. Every patient underwent an adapted diet calculated by the dietitian, considering individual needs and preferences, sometimes with formula supplementation administered through the tube. No standardized diets were applied to PEG patients by routine.

All participants or their representatives signed the informed consent. Patients with oncologic disease, quantified food ingestion above 50% of energy daily needs during the last month before PEG, evident inability to provide credible anamnestic data, or those who refused to participate in the study were excluded. Additionally, patients with diabetes mellitus and other clinical conditions (e.g., celiac disease, peptic ulcer, inflammatory bowel disease, and any endocrine disorder) or taking specific medications that could interfere with intestinal mucosa hormonal physiology were not included. The technical impossibility of obtaining duodenal biopsies and the expected inability to have an appropriate clinical and nutritional follow-up were additional exclusion criteria.

Random healthy individuals that underwent upper gastrointestinal endoscopy to investigate ferropenic anemia also collected duodenal biopsies, being used as normal controls if no endoscopic and histologic abnormality was diagnosed, and a non-duodenal source of bleeding was found.

### 2.3. Anthropometric Evaluation

Anthropometric evaluation was performed by a dietitian before the procedure, according to the *ISAK Manual of International Society for the Advancement of Kinanthropometry* [13]. The average of three consecutive measurements was recorded.

The body mass index (BMI) was obtained in most patients using the equation Weight/Height^2^, measured in kilograms and meters, respectively. If patients were unable to easily stand up for weight and height evaluation, BMI was estimated using the Mid Upper Arm Circumference (MUAC) and the regression equations described by Powell–Tuck and Hennessy, which were previously proved to provide a reliable BMI estimation in PEG-fed patients [14,15].

MUAC was measured in centimeters, using a flexible measuring tape wrapped around the mid upper arm, halfway between the olecranon and the acromion process.

According to the GLIM (Global Leadership Initiative on Malnutrition) criteria for diagnosing malnutrition [16], all patients presented at least one etiologic criteria—reduced food intake. So, patients were classified having malnutrition if they also presented a phenotypic criterion: low body mass index (BMI below 20 kg/m^2^ if under 70 years of age or BMI below 22 kg/m^2^ if 70 years-old or older), or significant weight loss (above 5% within the last 6 months or above 10% beyond 6 months).

### 2.4. Laboratory Evaluation

A blood sample was obtained just before the gastrostomy procedure. Serum albumin, transferrin, total cholesterol, and electrolytes, namely phosphorus, magnesium, and potassium, were measured at baseline (T0) and during follow-up (T1) as part of patient global nutritional and metabolic evaluation. Values of albumin below 3.5 g/dL, transferrin below 200 mg/dL, and total cholesterol below 160 mg/dL were considered low values, suggestive of malnutrition and/or poor prognosis. Normal cut-off values for serum electrolytes were considered, according to the laboratory institutional protocol, as follows: phosphorus: 2.5–4.8 mg/dL; magnesium: 1.5–2.1 mg/dL; and potassium: 3.5–5.0 mmol/L. Patients with low levels of these serum electrolytes would not start enteral refeeding until replacement and complete normalization.

### 2.5. Duodenal Biopsies Analysis

All paraffin-embedded tissue blocks were processed and four cuts per block with a three-micrometer thickness were performed. Tissue expression of CgA, GLP-1, GIP, and neurotensin were studied through an immunohistochemical analysis in both study moments. Briefly, antigen retrieval was performed by microwave heating in 10 mM citrate buffer (pH 6.0). Endogenous peroxidase was blocked using 3% hydrogen peroxide for 20 min, followed by a 30-min blocking step with normal serum. Slides were incubated overnight at 4 °C in 5% BSA with the following antibodies: CgA (1:200, ab17064, Abcam, Cambridge, UK), neurotensin (1:5000, 3488-7), GIP (1:500, ab30679, Abcam), and GLP-1 (1:4000, ab22625, Abcam). Sections were then incubated for 30 min with biotinylated polyclonal secondary antibodies (1:200; EO35301-2 or EO35401-2, Dako, Glostrup, Denmark), followed by a 30-min incubation with the avidin–biotin complex (ABC, 1:100 dilution in 5% BSA; Vector Laboratories, Peterborough, UK). 3,3′-diaminobenzidine (DAB; Dako) was used as chromogen, with the incubation times of 2 min for CgA, 30 s for GIP and GLP-1, and 10 s for neurotensin. All tissue sections were counterstained with Mayer’s hematoxylin.

Slides stained by immunohistochemistry were scanned and the acquired images were processed using ImageJ 1.54p software (National Institutes of Health), incorporating a color deconvolution plugin to isolate the stained regions from the background, enabling quantification of the specific area stained by the respective antibodies. The ratio of GIP, GLP-1, or NT to CgA percentage-stained area was calculated as an estimate of the proportion of the respective positive cells within the overall enteroendocrine cell population.

The percentage of stained tissue in the total tissue area was analyzed in every patient at baseline and after PEG refeeding. All findings were compared with duodenal samples of controls.

### 2.6. Statistical Analysis

The statistical analysis was performed using the Statistical Package for Social Sciences (IBM SPSS^®^ Statistics, version 29.0). Continuous variables were expressed as the mean and standard deviation or medians and interquartile ranges. Categorical variables were reported as total and relative frequencies. Normality was assessed using the Kolmogorov–Smirnov test. The Mann–Whitney test was applied to compare the quantification of hormone tissue expression between patients and controls. The evolution of hormone expression in patients during both study periods was assessed using the Wilcoxon signed-rank test. The general linear model was also used for multivariate analysis in order to investigate the association between the quantified hormone tissue expression and other clinical data such as patient survival.

## 3. Results

### 3.1. Patients

A total of 30 patients that fulfilled the inclusion criteria were initially included in the study: 16 men and 14 women, aged between 38 and 87 years (mean 67.1 ± 13.5). Thirteen patients (43.3%) were below 70 years. The main characteristics of the population are described in Table 1. The clinical indication for PEG was prolonged dysphagia due to amyotrophic lateral sclerosis (90%), post-stroke (6.7%), and esophageal motility disturbance (3.3%). All patients were eligible for PEG-feeding without previous nasal tube feeding due to chronic disease with anticipated prolonged dysphagia. Of 30 included patients, 14 completed the protocol at the two time periods (46.7%), 9 died before the end of the study (30%), 1 removed the PEG tube due to dysphagia resolution (3.3%), and 6 were lost for follow-up (20%). Of patients who died during follow-up, the median survival was 5.5 months. There were no major post-PEG adverse events, and mortality was not attributed to PEG-related complications in any patient. All deaths were caused by progression of the underlying disorders.

A total of 10 citizens (3 men and 7 women) aged between 42 and 77 years (mean 52.7 ± 11.3) who underwent upper gastrointestinal endoscopy with duodenal biopsies to investigate ferropenic anemia were included in the control group. All of them presented normal endoscopic and histologic duodenal evaluation and no cause for anemia was detected in upper gastrointestinal tract.

### 3.2. Nutritional Assessment: GLIM, NRS 2002, Anthropometry, and Laboratorial Data

All patients presented major reduction of oral ingestion and significant weight loss, allowing the diagnosis of malnutrition according to the GLIM criteria. NRS 2002 was ≥3 points in all patients, signaling high nutritional risk.

BMI before PEG ranged from 14.5 to 27.9 kg/m^2^ (mean 21.56 ± 3.5 kg/m^2^) and was considered low in 12 patients (40%) after being adjusted to the age group. On the day of gastrostomy, mean albumin was 4.1 ± 0.5 g/dL, transferrin was 220.2 ± 42 mg/dL, and total cholesterol was 173 ± 37 mg/dL, being considered low in 1 (3.3%), 9 (30%), and 8 (26.7%) patients, respectively. Although albumin, transferrin, and total cholesterol are dependent on several factors, low serum levels together with reduced ingestion, weight loss, and low BMI supported a malnutrition diagnosis at baseline in all of the patients. Serum electrolytes were normal at the time of PEG in all patients. BMI and laboratory parameters did not change significantly after PEG tube feeding (*p* > 0.05).

Nutritional assessment data are highlighted in Table 1.

### 3.3. Duodenal Mucosa Immunohistochemistry

**Figure 1:**
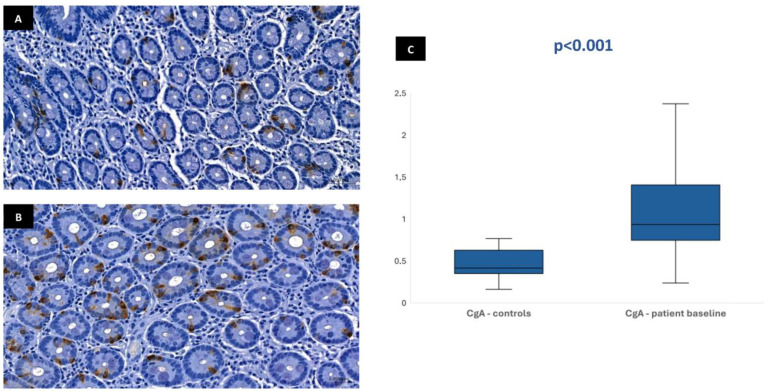
Duodenal mucosa histology with IHC analysis for chromogranin-A (magnification 34.1×), showing tissue coloration in the control group (**A**) and patients at baseline (**B**). The boxplot (**C**) describes both groups, showing significant differences.

**Figure 2:**
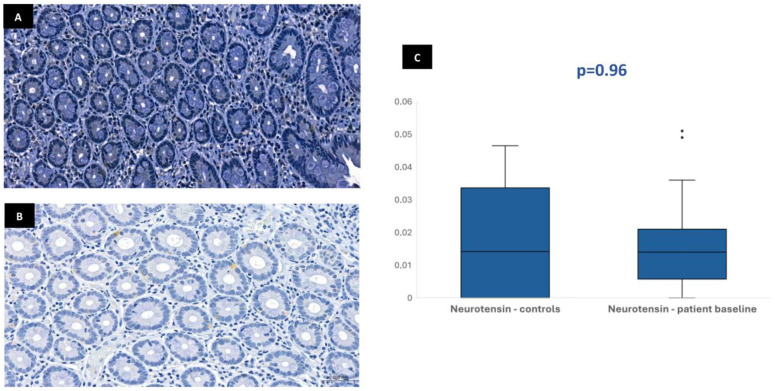
Duodenal mucosa histology with IHC analysis for neurotensin (magnification 34.1×), showing tissue coloration in the control group (**A**) and patients at baseline (**B**). The boxplot (**C**) describes both groups, showing no significant differences.

**Figure 3:**
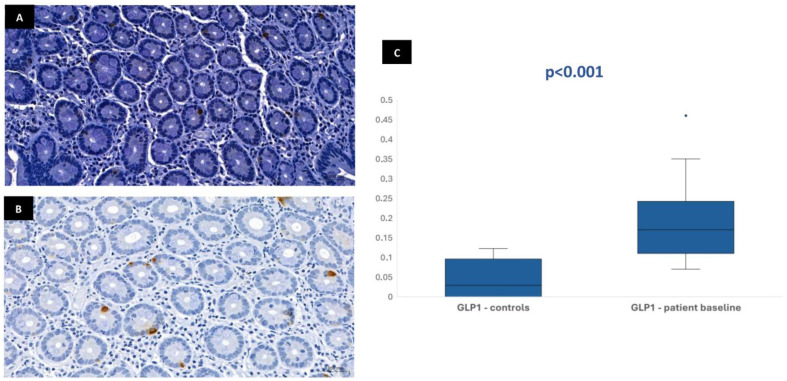
Duodenal mucosa histology with IHC analysis for the incretin GLP-1 (magnification 34.1×), showing tissue coloration in the control group (**A**) and patients at baseline (**B**). The boxplot (**C**) describes both groups, showing significant differences.

**Figure 4:**
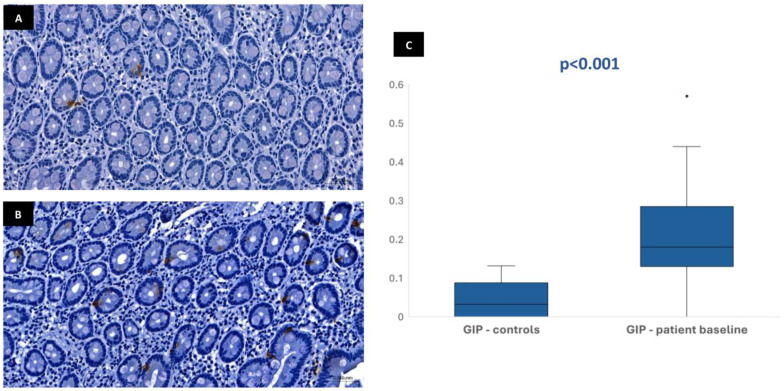
Duodenal mucosa histology with IHC analysis for the incretin GIP (magnification 34.1×), showing tissue coloration in the control group (**A**) and patients at baseline (**B**). The boxplot (**C**) describes both groups, showing significant differences.

In most of the 30 patients initially included in the protocol, it was possible to perform the immunohistochemical analysis at baseline: 29 patients for CgA, 28 patients for GLP-1, 28 patients for GIP, and 26 patients for neurotensin. This variation in sample numbers occurred because suitable histological tissue was not available for all markers in every case, either due to insufficient biopsy material or high background staining that did not allow a clear color isolation and reliable quantification of the immunohistochemical staining. Median stained area was 1.04% [IQR 0.75–1.47] for chromogranin-A, 0.17% [IQR 0.11–0.23] for GLP-1, 0.19% [IQR 0.13–0.32] for GIP, and 0.01% [IQR 0.01–0.02] for neurotensin. In the control group, the median stained area was lower for most hormones: 0.41% [IQR 0.35–0.63] for chromogranin-A, 0.03% [IQR 0–0.1] for GLP-1, and 0.03% [IQR 0–0.09] for GIP. Nevertheless, it was similar for neurotensin—0.01% [IQR 0–0.03]. Tissue expression of chromogranin-A, GLP-1, and GIP in patients at baseline was significantly higher than in controls (*p* < 0.001), but no difference was found for neurotensin (*p* = 0.96) (Figure 1, Figure 2, Figure 3 and Figure 4). No association was observed between baseline tissue expression of any specific gastrointestinal hormone with age, gender, BMI, laboratorial data, and patient survival in a multivariate analysis (*p* > 0.05).

After PEG tube feeding, duodenal mucosa immunohistochemistry could be assessed in 12 patients. In 2 patients, tissue samples were not adequate for immunohistochemical analysis. The median stained area was 0.88% [IQR 0.74–1.02] for chromogranin-A, 0.16% [IQR 0.12–0.19] for GLP-1, 0.19% [0.11–0.19] for GIP, and 0.01% [0.01–0.02] for neurotensin. No significant differences were observed between the two periods (*p* > 0.05)—Table 2.

## 4. Discussion

Protein-energy malnutrition is highly prevalent in gastroenterology clinical practice, especially when food intake is severely reduced for long periods. This condition, which is also exacerbated by disease-related inflammation, is commonly observed in patients undergoing PEG [3].

Prolonged fasting promotes several hormonal adaptative responses similar to stress-induced hypercatabolism, which includes increased levels of cortisol, downregulation of the hypothalamic–pituitary axis, high plasma catecholamine concentration, and decreased insulin/glucagon ratio, which increase lipolysis, proteolysis, and ketogenesis [17]. When malnourished patients are re-exposed to nutrition, the metabolism switches back to carbohydrates as the main source of energy in response to a serum insulin peak concentration. This sudden anabolism reactivation may lead to RS [18]. Although RS has been described in high-risk patients under total parenteral nutrition, especially when high caloric regimens are prescribed, some authors report an increased risk in patients undergoing enteral nutrition due to an increased serum insulin response induced by gastrointestinal incretins (GLP-1 and GIP) [19]. These peptides, which have been implicated in several pathogenic mechanisms, also deserve careful attention regarding starvation and refeeding physiology.

In the current study, the authors analyzed patients referred for PEG with significant low caloric intake and absence of previous nutritional support by tube feeding, looking for the mucosa tissue expression of gastrointestinal hormones including the incretins GLP-1 and GIP, CgA, and neurotensin. A large number of patients were diagnosed with amyotrophic lateral sclerosis (ALS), as significant reduction in oral ingestion was commonly observed during the early stages of this neurodegenerative disease [20]. Cancer patients usually present significant states of systemic inflammation and are submitted to cytotoxic therapies that would change intestinal homeostasis, and therefore, their inclusion was avoided. According to GLIM criteria, all patients presented malnutrition. Furthermore, almost 40% presented low BMI, an important malnutrition sign, which was consistent with the literature. Nevertheless, most laboratorial parameters were normal with less than 30% of patients presenting low serum proteins and total cholesterol, which could be explained by the reduced contribution of systemic inflammation to develop malnutrition in the early states of ALS, in stabilized post-stroke status and in esophageal functional dysphagia.

A histomorphometric analysis performed by our group in a previous reported study using the same patient samples revealed that more than two-thirds of starved patients presented median villi length below 0.5 mm being significantly shorter compared to controls. Additionally, enterocytes displayed changes in the striated plate with disarrangement of microvilli and increased autophagy demonstrated by the presence of cytoplasmic vacuoles. An increased length of intercellular spaces and cell detachment of basal membrane suggested compromised absorption. All these changes were not observed in controls and seem to be reverted after 3–6 months of enteral nutrition with an adequate intake [6].

The immunohistochemical evaluation of duodenal biopsies in the current study showed that mucosa expression of CgA, GLP-1, and GIP were significantly higher in patients compared with controls, suggesting an increased intracellular concentration of these substrates induced by prolonged fasting and malnutrition. Incretin secretion is stimulated by the presence of nutrients in the duodenal lumen, aiming to improve pancreatic insulin production for anabolism. The serum incretin concentration is typically low during fasting and increases immediately after eating [10,11]. The increased GLP-1 and GIP tissue expression found in our fasted patients seems paradoxical and could be viewed as an adaptative response of the duodenal mucosa—in a clinical setting of lower secretion stimulus due to reduced volume of intraluminal nutrients, increased mucosal incretin production could accomplish the need to maintain insulinogenic activity for essential anabolic processes. Additionally, incretin tissue expression may reflect the intracellular stores that need to be replenished during fasting for immediate release in circulation after food ingestion. Given the wide range of physiological processes and hormonal regulation in which CgA is involved, duodenal mucosa overexpression of this peptide may also contribute to maintain overall metabolic homeostasis regardless of low nutrient absorption. Since CgA also plays a role in the regulation of pancreatic secretion in response to enteral feeding, the higher tissue expression of this peptide in fasting patients, like what occurs with incretins, seems justified. Nevertheless, no significant differences regarding neurotensin were evident between patients and controls, possibly because of its very low overall tissue expression in both groups and the most significant role of this hormone outside the gastrointestinal system.

After 3–6 months of enteral refeeding, CgA and GLP-1 expression present a minor reduction, approaching the values of the control mucosa but without statistical significance. GIP and neurotensin did not present any change. Unlike what it was verified in the previous histological and ultrastructural analysis, duodenal tissue expression of incretins and neuroendocrine peptides in patients did not normalize after enteral nutrition during 3–6 months, suggesting that this time is probably insufficient to correct the hormonal adaptative responses induced by prolonged fasting. Actually, enterochromaffin cell turnover is much slower than the rapid turnover of the enterocytes, which can explain the potential long time needed for the normalization of hormonal expression in the duodenal mucosa after refeeding. Despite the adaptative changes observed in the duodenal histology and ultrastructure that suggest loss of absorptive function, which seems to protect patients from RS in the early post-PEG period, the increased tissue expression of incretins may otherwise contribute to exacerbating this risk. Although absorption is compromised in prolonged fasting patients, the incretin effect obtained after enteral refeeding and responsible for the insulin peak would probably be increased considering the high baseline expression of these hormones induced by chronic low ingestion. These findings associated with the long turnover of enterochromaffin cells of 16–150 days already reported may contribute to explaining the impact of RS beyond the first days of enteral nutrition, which was also addressed in a retrospective study previously reported by our group, where RS markers were shown to influence patient mortality during the first month of PEG-feeding [5,21].

Nevertheless, the present study displays some minor limitations that need to be considered. Thirty patients were initially included and assessed at baseline; however, only 14 completed the follow-up, and duodenal biopsies could be reanalyzed after refeeding. This occurred because one-third died before endoscopic control due to disease evolution. One patient recovered from dysphagia, and six were lost during follow-up. This may have influenced the interpretation of our results after PEG refeeding given the lower number of duodenal samples in the second period of the study. Further studies recruiting more patients during a longer period of follow-up will probably overcome this bias. Additionally, tissue overexpression of CgA, GLP-1, and GIP obtained in the immunohistochemical analysis compared to controls was interpreted as a surrogate marker of higher cellular activity and function of these proteins in fasting duodenal mucosa, which is debatable. Serum metabolic biomarkers for mucosa absorption and enteroendocrine cell function are required and deserve future research. Also, more studies to analyze the variation of serum concentration of these hormones during fasting and refeeding using mixed meal tolerance tests can probably also complement our results and clarify the association between tissue expression and biological activity of these substances. Finally, this study only addresses changes in the duodenal mucosa during fasting and immediate refeeding and does not assess if RS develops during follow-up from a clinical perspective.

In conclusion, the results obtained in this study suggest that prolonged fasting induces overexpression of duodenal mucosa gastrointestinal hormones and peptides assessed in an immunohistochemical analysis, which is associated with the already reported morphological changes. It included increased tissue expression of incretins and CgA as a potential adaptative mechanism to compensate for fasting-induced decreased absorption in order to maintain basal metabolism even under negative caloric supply. However, these changes may potentially increase RS risk after oral or enteral refeeding. As the present study also described, an immediate period of adequate enteral refeeding during 3–6 months was not sufficient to normalize these changes and to reestablish hormonal homeostasis in the duodenal mucosa. These findings may have potential implications for clinical practice as the risk of RS seems to be maintained despite chronic impairment to the intestinal mucosa. This occurs at the expense of hormonal metabolic rearrangements induced by prolonged fasting that persist longer due to enterochromaffin cell activity with slow turnover. The impact of these adaptative changes in RS management and clinical outcome may justify further research projects.

## Figures and Tables

**Table 1:** Baseline characteristics of patients.

Patient Characteristics		
**Age**	M = 67.1 ± 13.5 years	
**Gender**	Male: 16 Female: 14	(53.3%) (46.7%)
**Indication for PEG**	**Amyotrophic lateral sclerosis**: 27 **Post-stroke**: 2 **Esophageal dysmotility:** 1	(90%) (6.7%) (3.3%)
**Body Mass Index**	**Low**: 12 **Normal:** 18	(40%) (60%)
**Albumin**	**Low**: 1 **Normal**: 29	(3.3%) (96.7%)
**Transferrin**	**Low**: 9 **Normal**: 21	(30%) (70%)
**Total Cholesterol**	**Low**: 8 **Normal**: 22	(26.7%) (73.3%)
**Duodenal Mucosa Immunohistochemistry** **(% tissue stained area)**	**Chromogranin-A** **GLP-1** **GIP** **Neurotensin**	(1.04%) (0.17%) (0.19%) (0.01%)

**Table 2:** Evolution of gastrointestinal hormone tissue stained area at baseline and after PEG.

	Baseline (T0)	After PEG Feeding (T1)	*p*-Value
	Median Tissue Stained Area		
**Chromogranin-A**	1.04%	0.88%	0.94
**GLP-1**	0.17%	0.16%	0.36
**GIP**	0.19%	0.19%	0.31
**Neurotensin**	0.01%	0.01%	0.69

## Data Availability

The original contributions presented in this study are included in the article. Further inquiries can be directed to the corresponding author.
